# Coverage of Coronavirus Disease-2019 (COVID-19) Booster Dose (Precautionary) in the Adult Population: An Online Survey

**DOI:** 10.7759/cureus.26912

**Published:** 2022-07-16

**Authors:** Ramesh Masthi NR, Anagha Brahmajosyula, Aniket Khamar, Namita Acharya, Lavanya P Bilichod, Deepika Kondath

**Affiliations:** 1 Department of Community Medicine, Kempegowda Institute of Medical Sciences, Bangalore, IND

**Keywords:** covaxin, covishield, covid-19 vaccine india, covid vaccination booster, sars-cov-2, covid-19 vaccine efficacy, covid-19 india

## Abstract

Background

The coronavirus disease-2019 (COVID-19) pandemic devastated public health worldwide, including India. COVID-19 vaccines and their boosters are life-saving developments that have helped prevent and control the spread of COVID-19. We conducted this study to assess the coverage of the booster dose in an Indian population (the third dose of the COVID-19 vaccine in India is referred to as the booster or precautionary dose), record the reasons for not taking the booster dose, and determine the effectiveness of the booster. The levels of adherence to COVID-19 precautionary behavior was also assessed.

Methods

We conducted a descriptive, cross-sectional study using convenient sampling via an online survey of 550 respondents older than 18 in the second quarter of 2022. The respondents were distributed among 18 states and union territories in India. The data were analyzed as simple proportions and percentages.

Results

Of the 550 respondents, 152 (27.6%) received the booster dose, indicating low coverage. A small percentage of respondents (7.2%) reported suffering from COVID-19 following the booster, of whom 91% were medical professionals. The most common reported reason for not taking the vaccine was that the respondents were not yet due for their dose (48.1%). The time between the second dose of the COVID-19 vaccine and the booster had no impact on infection rates. Men were less likely to adhere to COVID-19 precautionary behavior than women, despite similar vaccination rates.

Conclusion

The COVID-19 vaccine booster had a low acceptance in our study population, with roughly one-quarter of the population receiving the booster. The booster dose has been influential in the prevention of COVID-19. Most respondents followed behavioral safety measures despite the decline of active cases of COVID-19 in India following the Omicron wave. Our results indicate a need to strengthen public strategies to affect behavioral changes, such as improving India's Behavior Change Communication program to ensure adequate booster dose coverage.

## Introduction

In December 2019, an outbreak of the novel coronavirus disease-2019 (COVID-19) caused by the severe acute respiratory syndrome coronavirus 2 (SARS-CoV-2) was initially detected in Wuhan, China. The first case in India was reported on January 27, 2020 [1]. Subsequently, it was declared a pandemic on March 11, 2020, by the World Health Organization [2]. In India, COVID-19 affected approximately 43 million people and has contributed to 524,706 deaths as of June 6, 2022 [3].

The COVID-19 vaccine was introduced in India on January 16, 2021, for the health care workers, frontline workers, and older patients (aged 60 years or older), and subsequently in different age groups in a phased manner [4]. The vaccines currently approved (as of June 6, 2022) for booster dose in India are CoviShield (AstraZeneca, manufactured by Serum Institute of India Private Ltd., Pune, India) and Covaxin (Bharat Biotech International Ltd., Hyderabad, India) [5]. Since the vaccines' introduction, 74% of the population has received the first dose, and 65% have received the second dose since June 6, 2022 [6].

A booster dose (i.e., a precautionary dose) was introduced in India for the health care workers, frontline workers, patients older than age 50, and those with comorbidities (Phase 1) on January 10, 2022 [7]. The Government of India announced the booster dose for patients aged 18 or older on April 10, 2022 [8]. The booster dose effectively prevented and controlled the spread of COVID-19 [9]. However, the decline in the number of active cases coincided with apprehension regarding the booster dose. Since the Omicron variant wave subsided in India, adherence to COVID-19 precautionary behavior (e.g., social distancing, mask-wearing, and hand hygiene) has declined in the population, significantly potentiated by the removal of mask mandates in several states [10]. Studies conducted during the previous waves in the Indian subcontinent showed persisting hesitancy among the general population regarding the COVID-19 vaccination [11].

It is imperative to assess the coverage of the COVID-19 booster dose and its relationship with the disease. Despite an extensive literature review, we found no studies of booster dose vaccinations as of this writing (June 6, 2022). However, similar studies conducted in other countries demonstrated the effectiveness of the booster dose in preventing severe COVID-19 and lesser rates of hesitancy for its uptake [12-14]. Therefore, we conducted this study to survey India's coverage/acceptance of the COVID-19 vaccine booster dose. The study also aimed to record reasons for not taking the booster dose and determine the effectiveness of the COVID-19 vaccine booster dose in preventing COVID-19. The study also measured the level of adherence to COVID-19 precautionary behavior.

## Materials and methods

We conducted this descriptive cross-sectional study in the second quarter of 2022. The study involved an online survey using Google Forms (Google, Mountain View, CA, USA) on respondents aged 18 years or older, living across the different states of India, representing a large geographical area. The sample size was calculated based on a pilot study coverage using the formula 4pq/r^2^, where prevalence (p) = 14 and q (1-p) = 86, and precision (r) = 3. The calculated sample size was 535, rounded to 550 respondents. We used a convenient sampling technique for the collection of responses. The investigators shared the questionnaire with their known contacts, who, in turn, shared it with their contacts until the sample size was met by snowball sampling. Only those respondents who had access to the internet and smartphones were part of the study.

The questionnaire consisted of 16 questions, semistructured with open- and closed-ended questions on the demographic information, vaccination status, booster dose, reasons for not taking the booster, history of SARS-CoV-2 infection, existing comorbidities, and adherence to COVID-19 precautionary behavior (see Appendix). Study participation was voluntary. All participants provided informed consent, and the Institutional Ethics Committee of Kempegowda Institute of Medical Sciences approved the questionnaire (reference number KIMS/IEC/A027/M/2022). Confidentiality was ensured so that only authorized persons had access to the data.

The results were analyzed appropriately using Google Spreadsheets (Google, Mountain View, CA, USA), and data were cleaned. The data were described in terms of median, percentages, proportions, graphs and Z-tests to determine significance. The data were considered statistically significant at p < 0.05.

## Results

A total of 550 respondents aged 18 or older were included in the study (275 men (50%); 272 women (49.5%); two respondents (0.3%) declined to disclose sex, and one (0.2%) was transgender). Most respondents (n = 238; 43.3%) were aged 18 to 30, 42 (7.6%) were aged 31 to 40, 166 (30.2%) were aged 41 to 50, 81 (14.7%) were aged 51 to 60, 23 (4.18%) were older than 60, and two (0.36%) were aged 81 or older. The median age of the respondents was 39 years.

The study received responses from 18 states and other union territories across India. Most respondents (n = 369, 67.1%) were from Karnataka; 46 responses (8.4%) came from Gujarat; 25 (4.5%) from Telangana; 22 (4%) from Maharashtra; 19 (3.5%) from Tamil Nadu; 18 (3.3%) from Kerala; 14 (2.5%) from West Bengal; seven (1.3%) from Uttar Pradesh; five (0.9%) each from Andhra Pradesh, Chhattisgarh, and Punjab; and two (0.4%) responses were from Haryana. Assam, Goa, Jharkhand, Madhya Pradesh, Rajasthan, and Sikkim had one (0.2%) response each. The remaining seven (1.3%) responses were collected from other parts of the country.

One hundred eighty-three respondents (32.9%) were graduates, 174 (31.63%) had a professional degree or were postgraduates, 175 (31.8%) were undergraduates, 16 (2.9%) were 12th pass/second pre-university course, and two (0.36%) had completed high school. Many respondents (n = 155, 28.2%) were medical, and allied professionals (e.g., doctors, students, nursing professionals, dentists, and paramedical professionals), and 395 (71.8%) were from various occupations in nonmedical sectors. Of the 155 medical and allied professionals, 125 (80.6%) were medical students, 24 (15.6%) were medical doctors, four (2.6%) were nursing professionals, one (0.6%) was a paramedic, and one (0.6%) was a paramedical student. Of the 395 nonmedical professionals, most (n = 68, 17.2%) were in information technology, civil engineering, automation, telecommunication, and aeronautics; 62 (15.7%) were homemakers; 55 (14%) were from the business sector; 50 (12.6%) were students (e.g., engineering, commerce); 38 (9.6%) were teachers/professors; 37 (9.4%) were salaried professionals; 12 (3%) were in the industrial sector; six (1.5%) were in the public services sector; five (1.2%) were in the marketing sector; 11 (2.7%) were from the legal and administrative sectors; five (1.2%) were architects; one (0.2%) was from arts sector; two (0.5%) respondents each were from the commerce industry, the agricultural sector, and media and journalism sector. 16 of the respondents (4%) reported to not working presently and 14 (3.5%) were reported to have retired. There were nine respondents (2.7%) from other nonmedical sectors. 

**Figure 1:**
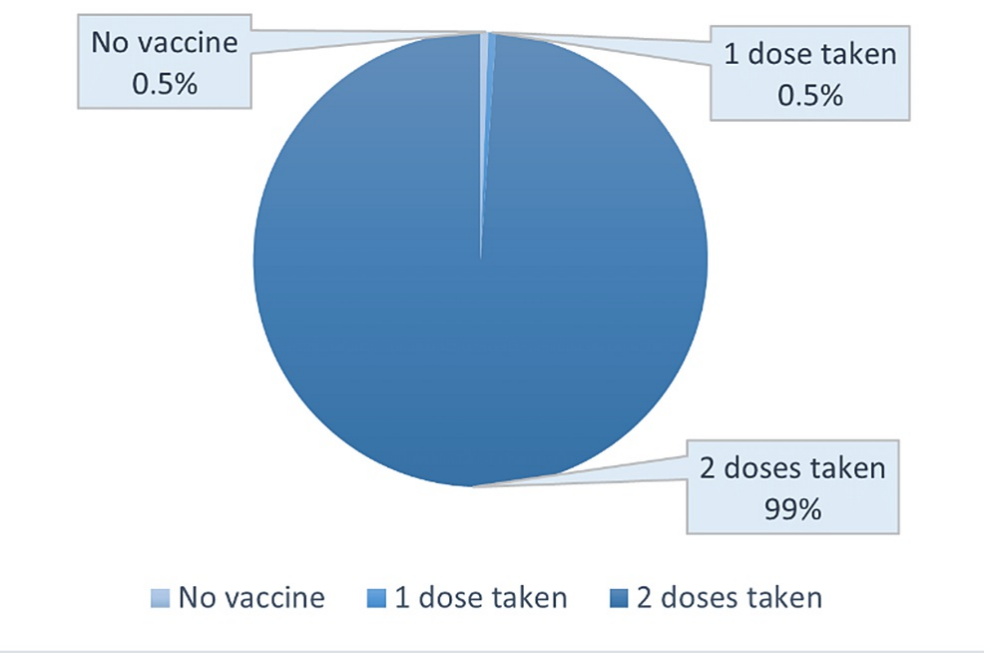
COVID-19 vaccine status of the respondents (n = 550)

Most respondents (n = 454, 82.5%) received the CoviShield vaccine as the primary dose, and 89 (16.3%) received Covaxin. Three (0.5%) received the Pfizer vaccine and two (0.4%) each received the Moderna and Sputnik vaccines. Nearly all respondents (544, 98.9%) reported receiving two doses of the COVID-19 vaccine, and only three (0.5%) respondents reported taking only one dose of the vaccine. The remaining three did not receive any vaccine doses (Figure 1).

Of the 152 (27.6%) respondents who received a booster dose, most (n = 75) were aged between 18 to 30. Only six respondents aged 31 to 40 reported receiving a booster dose (Table 1). 83 respondents (54.6%) were medical and allied professionals, and 69 (45.4%) were nonmedical professionals (Z = 8.434, p < 0.05). Thus, the booster coverage was 53.5% among medical and allied professionals and 17.5% among nonmedical professionals. Among the medical and allied professionals, 10 (12.0%) developed COVID-19 following the booster dose, and among the nonmedical respondents, only one (1.4%) developed COVID-19 afterwards. Of the 23 respondents aged 60 years and above, 18 (78.3%) received the booster dose, and none later developed COVID-19.

**Table 1:** Age distribution of the respondents with booster status (N = 550)

Age (years)	Booster Received, n (%)	No Booster Received, n (%)	Total N (%)
18-30	75 (31.5%)	163 (68.5%)	238 (100%)
31-40	6 (14.3%)	36 (85.7%)	42 (100%)
41-50	31 (18.7%)	135 (81.3%)	166 (100%)
51-60	22 (27.2%)	59 (72.8%)	81 (100%)
60 and above	18 (78.2%)	5 (21.7%)	23 (100%)
TOTAL	152	398	550 (100%)

Table 2 presents the time between the second vaccine dose and the booster. Most (n = 76, 50%) respondents received the booster nine to 12 months after the second vaccine dose, and 37 respondents (24.3%) reported receiving the booster six to nine months after their second dose of the vaccine.

**Table 2:** Comparison of respondents with booster dose and COVID-19 over time (n = 152) COVID-19: coronavirus disease-2019

Time Between Second Dose and Booster	Respondents, n	Respondents with COVID-19 After Booster, n (%)
1-3 months	6	-
3-6 months	13	1 (7.7%)
6-9 months	37	3 (8.1%)
9-12 months	76	6 (7.9%)
12-14 months	19	1 (5.3%)
> 14 months	2	-

Reasons for not yet receiving the booster are presented in Table 3. Most (n = 188, 48.1%) claimed they were not yet due for their booster dose. The next most cited reason (n = 41, 10.4%) was the apparent unavailability of the vaccine at the vaccine centers and the lack of slot availability on the COVID-19 Vaccine Intelligence Network portal [15]. The least cited reason (n = 2, 0.5%) was adverse events following the previous dose.

**Table 3:** Reasons for not taking the COVID-19 booster dose (n = 393) COVID-19: coronavirus disease-2019; AEFI: adverse events following immunization

Reason	Medical and Allied Professionals, n (%)	Nonmedical Professionals, n (%)	Respondents, n (%)	Z value (at p < 0.05)
Not yet due for the booster dose	29 (42.6%)	159 (54.6%)	188 (48.1%)	-4.792
Recent COVID-19 infection	8 (11.8%)	20 (6.9%)	28 (7.1%)	Not significant
Unavailable vaccine	9 (13.2%)	32 (11%)	41 (10.4%)	Not significant
Doesn't believe in the effectiveness of the vaccine	10 (14.7%)	14 (4.8%)	24 (6.1%)	Not significant
COVID-19 doesn't exist now	4 (5.9%)	11 (3.8%)	15 (3.8%)	Not significant
No trust in the government	1 (1.5%)	1 (0.3%)	2 (0.5%)	Not significant
Vaccine center is too far away	4 (5.9%)	2 (0.7%)	6 (1.5%)	2.107
Unaware about the booster dose	3 (4.4%)	26 (9%)	31 (7.9%)	-2.193
Felt that the booster is not necessary presently	-	6 (2%)	6 (1.5%)	Not significant
AEFI	-	2 (0.7%)	2 (0.5%)	Not significant
Will take the vaccine soon	-	18 (6.2%)	18 (4.5%)	-2.703
Others			32 (8.1%)	Not significant
TOTAL:	68 (100%)	291 (100%)	393 (100%)	

254 respondents (46.2%) reported receiving a COVID-19 diagnosis (confirmed by reverse transcriptase-polymerase chain reaction testing) in the past with or without vaccination. Among them, 73 (29.7%) stated that they had COVID-19 before receiving a COVID-19 vaccine dose, 36 (14.6%) had COVID-19 after the first dose, 126 (51.2%) after the second dose, and 11 (4.5%) of them reported to have COVID-19 after taking the booster dose. Among the 73 respondents who had COVID-19 before receiving their first vaccine, 17.8% received the booster dose. Of the 36 respondents who had COVID-19 after their first dose, 22.2% received the booster dose. Of the 126 who had COVID-19 after the second dose, 29.3% received the booster dose.

Of the 11 respondents who had COVID-19 following the booster dose, 10 (91.0%) were medical professionals, four medical doctors, and six medical students. One of the 11 (9.1%) received the booster dose within three-to-six months of the second dose, three (27.3%) received it within six-to-nine months, six (54.5%) received it within nine-to-12 months, and one (9.1%) respondent received the booster dose 12-to-14 months after the second dose. Only one (9.0%) nonmedical subject developed COVID-19 following booster administration.

121 respondents had associated comorbid conditions and other chronic illnesses. 31 (5.6%) had hypertension, 27 (4.9%) were obese/overweight, 24 (4.4%) had diabetes, seven (1.3%) had asthma, and the remaining had two or more comorbid/chronic illnesses in combination. 21 (17.4%) respondents with comorbidities were from the medical and allied sectors, whereas 100 (82.6%) were from nonmedical sectors. Among the respondents with existing comorbidities, 40 (33.1%) received the booster dose.

Of the 21 respondents who were medical and allied professionals with existing comorbidities, 12 (57.1%) received the booster dose, 10 of whom had a history of COVID-19. Nine respondents (42.9%) did not receive the booster dose, of whom five had a history of COVID-19. There was no statistical significance observed between the two groups that had and had not taken the booster dose. Of the 100 respondents from various nonmedical sectors, 28 (28%) received the booster dose. Among them, 15 had a history of COVID-19. 72 (42.9%) respondents did not receive the booster dose, and 36 had a history of COVID-19. There was no statistical significance observed. 

Of the 155 respondents from the various medical and allied professions, 136 (87.7%) claimed to continue following COVID-19 precautionary behavior (e.g., social distancing, mask-wearing, and hand hygiene). Of the 395 respondents from nonmedical fields, 361 (91.4%) followed COVID-19 precautionary behavior. However, there was no statistical significance between the groups. Of the 53 respondents who reported not following COVID-19 precautionary behavior, 24 (45.3%) were aged 18 to 30, 11 (20.8%) were aged 41 to 50, nine (17%) were aged 31 to 40, and nine (17%) were age 51 to 60. All respondents aged 61 and older reported following COVID-19 precautionary behavior. 12 (9.9%) of the 121 respondents with preexisting comorbid conditions/chronic illnesses reported not following COVID-19 precautionary behavior, while 41 (9.6%) of the 429 without existing comorbidities did not follow COVID-19 precautionary behavior. There was no statistical significance observed. 

Of the 11 respondents who had COVID-19 following the booster dose, three of them (27.3%) reported not adhering to COVID-19 precautionary behavior.

## Discussion

The COVID-19 vaccine was introduced in India in January 2021 for health care workers and frontline workers; those aged 18 and older were eligible for the vaccine in April 2021. Children and adolescents in India are not due for their booster dose because the COVID-19 vaccine's initial dose was introduced only in March 2022 for this age group [4,16,17]. COVID-19 vaccines are critical in preventing SARS-CoV-2 infection and severe COVID-19-related outcomes [18,19].

In January 2022, the booster dose (precautionary dose) was made available to high-risk individuals (e.g., doctors and frontline workers) to boost their waning immunity [7]. Booster eligibility was later extended to other groups. CoviShield and Covaxin are currently approved for booster doses in India [5]. Mixing COVID-19 vaccines was not allowed in India, meaning the precaution dose must be the same as the first and second doses during the study [20].

Most respondents received CoviShield, which is not surprising because CoviShield is the predominant vaccine administered in India, covering 80% of the market share [21]. Most respondents received two doses of the COVID-19 vaccine, while only 28% received the booster dose. The higher coverage in the present study was observed because the respondents were predominantly in medical professions. However, coverage of the booster in India among the general population is reported to be only 2.5% as of June 6, 2022 [6].

While medical professionals had higher uptake of the booster dose (more than 50%) than the nonmedical respondents (<20%), we found that nearly half of surveyed medical professionals have yet to take the booster dose. A significant number of medical professionals stated that they were not yet due for their booster, which was surprising because the first and second doses of the CoviShield vaccine had to be taken one month apart as per the initial guidelines for the frontline workers [22]. This could be attributed to the general hesitancy to take the vaccine at the time of its announcement due to which the subsequent administration of the booster dose was unexpectedly delayed even among medical professionals [23]. However, further analysis regarding the same could not be conducted as the data on when each respondent received the initial doses of the COVID-19 vaccines were not collected. 

Among the rest of the respondents from medical and allied professions who did not receive the booster, some did not believe in the efficacy of the vaccine, some did not believe that COVID-19 existed anymore, some claimed that the booster dose was unavailable, and others were entirely unaware of the announcement of the booster dose. All these reported reasons point to the failure of India's Behavior Control Communication (BCC) strategies regarding the COVID-19 vaccination program. The BCC is an integrated and interactive process with communities to develop tailored messages and approaches using a variety of communication channels to develop positive behaviors; promote and sustain individual, community, and societal behavior change; and maintain appropriate behaviors regarding COVID-19 safety. India has implemented various strategies such as the BCC to ensure public awareness of COVID-19 and its vaccines [24]. 

Most of the remaining respondents (mostly nonmedical professionals) claimed that they were not yet due for their booster dose, but this could be because the booster dose was announced only one month prior to our survey (April 10, 2022) for individuals aged 18 or older in India [8]. Thus, hesitancy may not have been the main reason for the low coverage of the booster dose in the public. This contrasts with the previous doses of the vaccines, where hesitancy was the main governing factor for the low vaccination rates [11]. Another reason for the lower uptake of the booster dose among the nonmedical professionals than medical and allied professionals could be better awareness of their occupational risk and coexisting comorbid status, generating a greater perceived need by the medical professionals. Other factors influencing the lesser coverage of the booster in nonmedical professionals could be the general misinformation regarding the time gap between the administration of booster and the second dose of the COVID-19 vaccines and reduced fear of the disease due to the plateauing of cases in the country following the Omicron wave [25,26].

The percentage of respondents who developed COVID-19 following the booster dose were similar regardless of the time interval between the second dose and the booster dose as presented in Table 2. Thus, we conclude that there was no significant impact due to the time of administration of the booster dose (with respect to the second dose) on the rates of development of COVID-19. The Government of India's move to set the eligibility for booster dose administration as nine months or later appears to be a sound strategy as the interval following the second dose has not impacted the occurrence of SARS-CoV-2 infection [7].

Following the booster dose, only 11 respondents developed COVID-19, most of whom were from medical and allied sectors. Furthermore, among the medical and nonmedical professionals who received the booster dose during the same period, the rate of development of COVID-19 was higher among medical and allied professionals. Therefore, the significant occupational risk of medical professionals was the leading cause of COVID-19 following the booster dose among these respondents [27].

The current study revealed that medical professionals were slightly less inclined (12.1%) to follow COVID-19 precautionary behavior than professionals from other sectors (8%). However, there was no statistical significance observed for the same. 

Our study had several significant limitations. The main limitation of this study was that only urban and educated parts of Indian society with access to technology could access the survey and participate, meaning there was an inherent selection bias with the sampling technique. Performance bias was also another significant limitation. Larger studies covering the entire country with equal representation of all the states would provide deeper insights into the situation. As the booster dose was announced for the general population only one month prior to our survey, it is imperative to conduct future studies to reassess the situation of booster uptake in the country. 

## Conclusions

The coverage of COVID-19 booster doses was low among the respondents, including medical professionals. The booster dose has been effective in preventing COVID-19. Most respondents followed COVID-19 precautionary behavior despite the decline of active cases of COVID-19 in India following the Omicron wave. Our results indicate a need to strengthen public strategies to affect behavioral changes, such as improving the Indian BCC program, to ensure adequate coverage of the booster dose.

## Appendices

The survey instrument used in the study

This is an online survey entitled: "Coverage of COVID-19 Booster (Precautionary) Dose in the Adult Population" being conducted by a group of 4th-year medical students from Kempegowda Institute of Medical Sciences, Bangalore. We intend to study the public acceptance for the COVID-19 precautionary dose vaccines. The survey will take around 5 to 10 minutes of your time.

The questions are related to the demographics of the respondents and their familiarity with the COVID-19 vaccination.

If you have any queries related to the questionnaire- please feel free to contact the undersigned.

We thank you for your time and cooperation!

Anagha Brahmajosyula

Aniket Khamar

Deepika Kondath

Lavanya Bilichod

Namita Acharya

- 4th year MBBS, Kempegowda Institute of Medical Sciences, Bangalore.

INFORMED CONSENT:

PARTICIPATION

Your participation in this survey is purely voluntary. We assure you, your identity would be strictly anonymous and the information provided in the questionnaire would be used only for academic purposes.

* Required 1. Gender * (Mark only one oval.)

o    Male

o    Female

o    Transgender

o    Prefer not to say

2. Age * (Mark only one oval.)

o    18-30

o    31-40

o    41-50

o    51-60

o    61-70

o    71-80

o    81 and above

3. Education * (Mark only one oval.)

o    Professional degree (PhD scholar) / Postgraduate

o    Graduate

o    Undergraduate

o    12th pass/ 2nd PUC

o    High school (8th to 10th grade)

o    Middle school (6th to 8th grade)

o    Primary school (1st to 5th grade)

o    Other:

4. Location (this form is applicable only for those living in India) * (Mark only one oval.)

o    Andhra Pradesh

o    Arunachal Pradesh

o    Assam

o    Bihar

o    Chhattisgarh

o    Goa

o    Gujarat

o    Haryana

o    Himachal Pradesh

o    Jharkhand

o    Karnataka

o    Kerala

o    Madhya Pradesh

o    Maharashtra

o    Meghalaya

o    Mizoram

o    Nagaland

o    Odisha

o    Punjab

o    Rajasthan

o    Sikkim

o    Tamil Nadu

o    Telangana

o    Tripura

o    Uttar Pradesh

o    Uttarakhand

o    West Bengal

o    Others: Please mention

5. Which city do you live in? (Please mention) *

6. Occupation * (Mark only one oval.)

o    Medical student

o    Paramedics

o    Paramedical student

o    Student (engineering, commerce, etc.)

o    Business sector

o    Agriculture, animals, environmental sector

o    IT, civil engineering, automation, telecommunication, aeronautics

o    Education (Teacher/Professor)

o    Industrial workers, production, manufacture

o    Housekeeping and cleaning industry

o    Driver, Transport industry, Aviation industry

o    Housewife

o    Public service/ government job

o    Media, journalism, graphics, printing, design

o    Marketing, PR, advertising

o    Legal, Administration

o    Hospitality, tourism, leisure, sports

o    Commerce industry

o    Architecture, interior decorators

o    Arts industry

o    Salaried professional

o    Nursing/Allied Health Sciences/Dentistry

o    Retired/not currently working (medical and allied professionals - doctors, paramedics, dentists, nursing professionals.)

o    Retired/not currently working (nonmedical professionals)

o    Others

7. Have you taken the COVID-19 Vaccine? * (Mark only one oval.)

o    No vaccine taken

o    1st dose taken

o    2 doses taken

8. Which vaccine have you taken? (Mark only one oval.)

o    CoviShield (Oxford AstraZeneca)

o    Covaxin

o    Sputnik V

o    Pfizer

o    Moderna

o    Other:

9. Have you taken the booster dose? * (Mark only one oval.)

o    Yes

o    No

10. If yes, how many days after the 2nd dose did you take the booster dose? (Mark only one oval.)

o    1-3 months

o    3-6 months

o    6-9 months

o    9-12 months

o    12-14 months

o    Greater than 14 months

11. If no, why? (i.e., If you have not taken the booster dose) (Check all that apply.)

o    Not yet due for the booster dose

o    Recent COVID infection so you don't believe you need the vaccine

o    Unavailable vaccine

o    Doesn't believe in the effectiveness of vaccine

o    Believes that COVID-19 doesn't exist anymore

o    No trust in the government

o    Economic reasons

o    Vaccine center is too far away

o    Unaware about the booster dose

o    Adverse effects with any previous dose

o    Other:

12. If you have taken the booster dose, how many days has it been since the booster dose has been given? (Mark only one oval.)

o    Less than a month

o    1 month

o    2 months

o    3 months

o    Greater than 3 months

13. Have you had a COVID-19 infection / COVID-19 like symptoms anytime in the past? * (Mark only one oval.)

o    No

o    Yes

14. If yes, when was it? (Mark only one oval.)

o    Before any vaccine

o    After 1st vaccine dose

o    After 2nd vaccine dose

o    After the booster dose

15. Have you been diagnosed with any of the following? * (Check all that apply.)

o    Diabetes

o    Hypertension

o    Overweight/ obesity

o    Asthma

o    None

o    Other:

16. Do you continue to follow COVID-19 precautionary behaviour? (i.e., wearing masks, using sanitizers, social distancing, etc.) * (Mark only one oval.)

o    Yes

o    No
